# The engagement of people with lived experiences in substance use research

**DOI:** 10.3389/fpubh.2025.1611836

**Published:** 2025-10-01

**Authors:** Catherine Kim

**Affiliations:** Faculty of Community Services, Toronto Metropolitan University, Toronto, ON, Canada

**Keywords:** people with lived experience (PWLE), trustworthiness, substance use, mental health, reflexivity

## Abstract

**Background:**

People with lived experiences (PWLE) are underrepresented in research engagement, however their involvement can significantly boost the relevance and impact of research. Questions concerning the credibility and trustworthiness of PWLE researchers by traditional and positivist researchers have been identified. Having been associated with substance use of questionable legality and related substance use activities, PWLE researchers face stigma and are deemed to lack the trustworthiness that serious research entails. Current literature on PWLE found a dearth of knowledge on the definitions and conceptualizations of PWLE in research, which this paper attempts to partially address.

**Methods:**

Issues surrounding the trustworthiness of PWLE in substance use research were investigated, along with accounts of involving PWLE at different phases of the research process.

**Findings:**

People with lived experiences have been undervalued as researchers compared to other positivist counterparts despite advocating against marginalization and oppressive practices. They offer in-depth, meaningful contributions to research involving phenomena that they have experienced and were found to provide insights that other non-PWLE researchers overlooked. Moreover, engaging PWLE in research is not only beneficial for research processes and outcomes but is also empowering for PWLE themselves.

**Conclusion:**

A guide to maintaining trustworthiness and a description of PWLE contributions to research processes are provided.

## Introduction

People with Lived Experience (PWLE) are individuals who have directly encountered specific conditions or situations, providing them with unique insights and understanding that others without such experience may lack [1]. This includes those who have gone through health challenges, social difficulties, or other significant life circumstances. Often referred to as “experts by experience,” PWLE possess knowledge rooted in their personal journeys rather than relying on second-hand information, education, or media sources (1, 2).

Incorporating lived experiences into research can significantly boost its relevance and impact, particularly in the fields of mental health, where conventional methods have overlooked the unique insights of PWLE (3). PWLE encompass a diverse group of individuals from varying backgrounds, who can inform research in fields involving their relevant experiences, by grounding research questions and findings in real-life experiences, making it more relevant and improving its quality by identifying factors that might otherwise be overlooked (4, 5). For example, PWLE are likely to have experienced situations specific to the phenomena in question, such as the uncertainty involved when pondering one’s own experience, understanding the impact of experiences over time, and embodying difficulties related to stress or other conditions (6). These characteristics can be challenging to understand for individuals and researchers who have not personally experienced the phenomenon, and as a result, they may be overlooked if people with lived experience (PWLE) are not involved in the research.

In recent years, the value of incorporating lived experience into research has become increasingly recognized, particularly in fields such as mental health and substance use (5, 7). For example, The Centre for Addiction and Mental Health in Toronto, Canada has created a useful guideline document for engaging PWLE in substance use research (4), and The National Institute on Drug Abuse in the United States have also committed to incorporating PWLE in its research as evident in its various initiatives and strategic plans (8).

A scoping review by Hawke et al. (9) found that incorporating PWLE in substance use research was seen to enhance various aspects of the research process, including the overall quality of research (such as increased rigor, trustworthiness, and community relevance), specific research activities (such as participant recruitment), and the research environment itself (for example, by shifting power dynamics). Further research by Hawke et al. (4) documented the barriers, facilitators, and best practice guidelines for engaging PWLE. What appears to be lacking in the current literature are effective strategies to reinforce trustworthiness when confronting potential stigma from non-PWLE researchers, as well as a clear outline of the importance of involving PWLE throughout every stage of the research process.

This paper seeks to explore perceptions of the trustworthiness of PWLE in substance use research and offers guidance on how to foster and support this trust. Concerns about trustworthiness and possible biases from non-PWLE researchers and professionals have prompted a closer look at ethical issues associated with PWLE participation, with particular attention to the risks of confirmation bias and undue influence (10, 11). Researchers in general are expected to practice reflexivity and remain mindful of their inherent inclination to prioritize information that aligns with their pre-existing beliefs, which can result in less attention being given to evidence that contradicts those beliefs (12). Indeed, PWLE have been found to maintain respectful and ethical approaches in handling sensitive and private data (5) while maintaining reflexivity throughout all research processes (6).

In addition to navigating concerns about trustworthiness, having to disclose experiences that may have previously resulted in negative consequences while engaging in substance use activities may cause personal distress, stigma, and the possibility of re-traumatization in PWLE (7, 13). Roennfeldt et al. (14) describe “*a Catch 22 of needing to be exposed to potential discrimination in order to ultimately shift prejudicial attitudes”* (p. 108), as PWLE have indicated their strong drive for reform in mental health care following their diagnosis and experiences of substance use (14).

It is essential to ensure that studies involving PWLE are conducted ethically and with critical attention to power dynamics, as emphasized in conceptual frameworks such as critical race and feminist methodologies (6, 15). Consistent with these methodologies, PWLE have been found to oppose marginalization and oppression (14), and many PWLE have been drawn towards research that aims to enhance social justice (14).

In addition to discussing issues concerning the trustworthiness of engaging PWLE in research, this paper provides an overview of PWLE contributions to each stage of the research process, underscoring the importance of their active involvement throughout, rather than engaging them only as symbolic participants. An examination of theoretical and methodological implications further underscores the significance of involving PWLE in research.

## Trustworthiness

Professionals in traditional, evidence-based medicine have been called out for marginalizing PWLE by reinforcing rigid knowledge hierarchies and neglecting systemic power inequities (5). Additionally, having been associated with substance use of questionable legality and related activities, PWLE face stigma from other non-PWLE researchers, and are deemed to lack the trustworthiness that serious research entails (10). However, concerns about potential for biases (11) and undue influence on participants (16) can be mitigated by the adoption of reflexive practices throughout the entire study.

For example, research in substance use has generally attributed addiction recovery in individuals to clinical intervention, however this does not account for all instances of remission (17). For example, the discontinuation of substance use can occur with apparent spontaneity in individuals, without intervention; behavior which may be difficult to categorize or understand with traditional methods of analysis (17). It is logical to involve PWLE in this research as they are regarded as experts due to their firsthand knowledge and experiences, and their inclusion can enhance research by providing valuable and diverse perspectives into such nuanced situations [13]. The rigor and quality of all research can be elevated by adopting and describing the use of key concepts related to trustworthiness that were outlined by Lincoln and Guba (18).

The following four dimensions have been proposed as key indicators of trustworthiness in qualitative research [(18), as cited in (19)]:

1. *Credibility* refers to the accuracy of the study, which can be achieved by engaging in persistent observation, extended involvement, and triangulation of findings (30). PWLE are considered to have high credibility due to their real-life experiences and in-depth understanding of the nuances involved in the experiences of substance use diagnosis and treatment (4, 5, 7, 13, 14, 17, 30).
  Triangulation of data from different methods, different theoretical approaches, and varying contexts can add credibility to the research by demonstrating rigor and in-depth analysis from various viewpoints.
  Negative (deviant) case analysis adds to credibility by challenging researcher biases and adding transparency to the research.
2. *Transferability* refers to the application of findings in similar contexts, and can be facilitated by providing comprehensive and detailed (thick) descriptions of each research setting. Thick descriptions may include useful information such as participants’ views and motives, as well as the psychological, institutional, sociological, and anthropological context of the research environment (20).
3. *Dependability* refers to the consistency of research results, which can be established by rigorously documenting the research process through the creation and maintenance of a detailed audit trail.
4. *Confirmability* refers to the transparency of results; that findings are determined by participant engagement rather than through researcher interference or bias. The practices of peer debriefing, member checking, and reflexive journaling encourage transparent linking of data with research findings [(18), as cited in (19)].

In 1989, Guba and Lincoln recognized *authenticity* as an essential concept which expanded their earlier criteria of trustworthiness. *Authenticity* emphasizes the fair and accurate representation of participants’ voices and realities, moving beyond methodological rigor to incorporate the ethical dimensions of inquiry (21). Roennfeldt et al. (14) found that PWLE researchers are committed to honoring the individual voices of participants, while engaging in reflexivity throughout the entire process (6). As researchers, PWLE recognize that the focus is not on their personal stories. They are expected to maintain the confidentiality of their own experiences and avoid potentially influencing participants’ choices regarding treatments or decisions. Indeed, PWLE bring a heightened understanding of reflexivity and self-awareness, which ensures that personal biases and experiences do not influence the research process (16).

Asserted that PWLE possess in-depth knowledge that others lack, therefore they have the highest expertise on the topic that should be afforded a top level of validity [as cited in (7)]. “*This is a kind of disease you can only comprehend if you have experience of your own. Nobody else understands what it means”* [as cited in (7)].

Critics argue that PWLE engagement lacks objectivity, but “*complete objectivity is neither possible nor ideal in research*” (14). When engaging in research, all researchers including PWLE should identify the trustworthiness strategies that they employ, in order to demonstrate their commitment to rigor throughout the study. This will promote the acceptance of their research findings (30) and enhance the transparency and critical evaluation of the research (19).

By embedding PWLE expertise and reflexivity at every stage—from honing research questions to knowledge dissemination—the research process upholds the key dimensions of trustworthiness: credibility, transferability, dependability, and confirmability in addition to authenticity. This comprehensive integration counters skepticism about PWLE objectivity and demonstrates their vital role in producing rigorous, ethical, and impactful substance use research.

## Engaging PWLE throughout all research processes

PWLE can be engaged in diverse ways during research, including as lead investigators, refining research questions, creating /advising on research design, analysis, ethics, developing methodologies, carrying out research activities, analyzing and interpreting data, facilitating knowledge translation, and indeed as participants themselves (4, 27). PWLE engagement has been shown to increase participant recruitment, inform service design, and enhance knowledge dissemination activities (5). In efforts to conceptualize PWLE involvement in research, major research processes are identified in Table 1 below, along with the contributions that PWLE can make to these processes.

**Table 1:** Conceptualization of research processes involving PWLE.

Research process	PWLE contributions
Research question development	Possess embedded knowledge, experience, and skills that researchers without similar backgrounds may lack Ensure relevance, priorities, and real-world focus of research Contribute with topics based on their lived experiences, including ideas that other researchers may not have considered^1^
Research design	Tailor interventions that are more inclusive and sensitive to the actual needs and preferences of end users Develop products, treatments, or services collaboratively which tend to gain greater trust and have a higher likelihood of being embraced by the communities they are intended to benefit^2^ Design research methods that are more likely to yield outcomes that have meaningful practical relevance and enjoy greater acceptance among the intended population^3^
Recruitment	Provide advice on effective and appropriate recruitment strategies and sample representativeness As individuals who shared similar experiences, instilled trust in potential participants and increased recruitment numbers^4^
Data collection	Create ethically-safe data collection tools^5^ that uphold participants’ rights and dignity while minimizing potential harm throughout the research process^13^ Made interviewees feel more comfortable than traditional researchers^6^ Enhanced the clarity of the interview questions to participants and supported academic researchers in accurately understanding what the participants aimed to communicate^7^ May use language that is understandable to everyone who is involved, thereby providing greater accessibility to the research than other healthcare professionals Bring a heightened understanding of self-awareness that counteracts any personal biases^11^ Can establish rapport with participants more easily, leading to richer, more nuanced data collection, as well as reduced potential for harm or discomfort during data collection^12^ Involvement challenges stigma and shifts power dynamics, empowering individuals who have historically been marginalized or silenced in healthcare settings^4^.
Data analysis	Contextualize data and interpret findings in ways that reflect real-world experiences and nuances, promoting credibility and maintaining grounding in reality^8^ During coding, can identify patterns and themes that others may overlook due to their in-depth experiential knowledge Confirm that research data align with actual participant experiences, thus increasing rigour^9^
Research outcomes	PWLE have a personal stake in the outcomes of their research, as the findings may directly impact themselves and their communities. This investment fosters a deep commitment to ensuring that research is conducted ethically and with the utmost care. The personal connection to the research topic also motivates PWLE to ensure that their work contributes positively to their communities Question biases and assumptions, safeguarding results from overlooking the voices of participants^10^
Knowledge translation	Bridge findings with real-world applications based on their firsthand experiences Contribute to wording that is understandable by those that will be most affected by the results Disseminate knowledge in ways that are practical and applicable to target populations and real-world contexts

^1^Simpson et al. (28); ^2^Thorburn et al. (29); ^3^ Beames et al. (3); ^4^ Sheikhan et al. (5); ^5^CCSA (13); ^6^Croft et al. (25); ^7^Moltu et al. (26); ^8^Stull et al. (17) and Prosek and Gibson (30); ^9^Ahmed (19); ^10^Roennfeldt et al. (14); ^11^Mental Health Research (16); ^12^ Berg et al. (23); ^13^ Lim (24).

PWLE engagement in all stages of research is essential because of the in-depth understanding and insight that they offer, while maintaining applicability to relevant participant populations. These processes, in conjunction with the trustworthiness indicators mentioned previously, highlight the utility of PWLE researchers by fostering ethical and respectful engagement with the data and participants. Overall, by engaging PWLE, research can become more inclusive, ethical, and impactful^3^.

## Implications

### Theoretical implications

There are several implications for engaging PWLE in research. First, PWLE engagement challenges positivist frameworks that tend to prioritize quantifiable, objective data over experiential, subjective knowledge (5, 13, 14). By integrating PWLE viewpoints, reality-grounded and pragmatic lenses are brought to the research (9), which challenge traditional hierarchies of knowledge.

Second, PWLE represent a minority voice (14), and they are uniquely situated to reveal experiences of marginalization and oppressive human rights practices that perpetuate stigma and discrimination (14). According to Roennfeldt et al. (14), PWLE are often drawn towards research methods that advocate for social justice, address power imbalances, and amplify individual voices. This approach embodies critical theories by identifying and dismantling traditional power structures and discriminatory practices in knowledge production, and actively empowering the perspectives of PWLE to reshape dominant narratives and methodologies (9).

Engaging PWLE enhances research not only by providing deeper, experiential knowledge of the phenomena being studied, but also by enriching the connection between the theoretical framework, knowledge translation, and practical application to relevant participants (3).

### Methodological implications

The engagement of PWLE in research is most relevant when attempting to gain in-depth knowledge of specific phenomena in the context of that phenomena (30). Depth of understanding is emphasized over wide-ranging breadth of knowledge, and purposive sampling will ensure relevance of the participants to the phenomena in question (30).

Engaging PWLE in research aligns with emancipatory methodologies that aim to reshape the processes of knowledge creation and data analysis. Experiential knowledge is grounded in real-world contexts, shifting emphasis from the use of quantitative methods of data collection to participatory paradigms and inductive analyses. Indeed, has found that the results of satisfaction surveys for substance use treatment often do not align with results obtained from qualitative methods, and research methods that involve PWLE can uncover deeper insight that are not captured in standardized assessments (30).

## Conclusion

The inclusion of PWLE has become increasingly recognized as vital to enhancing the relevance, impact, and quality of research (5, 7). Involving PWLE researchers will minimize wasted health research efforts caused by misaligned priorities and practical needs—estimated at 85% (3)—by committing to actionable, real-world outcomes rather than disconnected inquiries.

Despite concerns regarding the trustworthiness and potential biases of PWLE in research, PWLE were found to impart high levels of insight and experiential knowledge to every stage of substance use research, which can be supported with transparent reflexive and trustworthiness practices throughout.

Involving PWLE offers significant advantages for research while also fostering personal and professional growth for these individuals (5). PWLE are no different from others and have been shown to excel as leaders, researchers, and collaborators (5, 14). Their research insights are integral to questioning established theories, combating stigma, and creating services that address the varied needs of substance use populations. PWLE engagement can help dispel stereotypes about people who have formerly used substances and demonstrate that recovery is possible. This can counteract stigma and encourage such individuals to seek help without fear of judgment (5, 17).

A limitation of this paper is that it provides a discussion on PWLE, that is, people with *past* living experiences, rather than those who are currently *living* with the experiences in question. People with *Living* Experience are individuals who are currently using one or more substances and may or may not be in recovery, which is “*a complex multi-faceted process of moving towards improved health, well-being and quality of life*” (22). This paper focuses exclusively on People With Lived Experience due to the additional ethical considerations involved when working with individuals currently living with substance use. Such considerations may include concerns with current level of sobriety, the need for supervision, or support (however unplanned or unintentional this may be), accessibility and safety considerations, etc. Such ethical complexities are numerous and extend beyond the scope of this paper.

Despite this limitation, this paper highlights that PWLE contribute unique strengths that greatly improve the relevance, quality, and impact of research. Future research in mental health and substance use should prioritize the inclusion of PWLE on research teams to leverage their deep and extensive experiential knowledge, which will result in improved quality and outcomes of the research. This may involve tackling systemic barriers to inclusion and research funding that PWLE may be faced with. By valuing lived experience, we can promote more inclusive, effective, and ethical research practices that ultimately lead to better outcomes for the populations served.
